# Review of: *Interpreting and Reporting Clinical Trials – A guide to the CONSORT statement and the principles of randomised controlled trials* by Anthony Keech, Val Gebski and Rhana Pike

**DOI:** 10.1186/1745-6215-9-27

**Published:** 2008-05-19

**Authors:** Stephen JW Evans

**Affiliations:** 1 Medical Statistics Unit, London School of Hygiene & Tropical Medicine, Keppel Street, London WC1E 7HT

The author declares that they have no competing interests.

